# Progress in Dermatology

**Published:** 1901-09-28

**Authors:** 


### PROGRESS IN DERMATOLOGY.

**Lupus.**—Numerous improvements in the new physical methods of treatment are being brought forward. Concentrated light and the X-rays are utilised in fresh ways. Finsen's apparatus has been improved not only by himself, but by other workers. One of the new lamps shown at Cheltenham is much less costly, and employs only a 2,000-candle power burner for each patient. The area of skin treated is doubled and the time required for a sitting is only one-third of that formerly needed. Finsen and others now employ the X-rays to aid the process of cure because they can be used for many places, such as the mucous membranes, to which the light rays are inapplicable, and because they act more quickly in certain stages of the disease and are less expensive to work. Geyser,[1] of New York, recommends the rays to be applied for seven to ten minutes and to be followed by the static spray to relieve any pain caused. Sunlight is falling into disuse, partly from its uncertainty in this climate. Werther[2] reports four cases treated by hot air at 300° while the patients were under an anæsthetic, and claims that three of them were cured Dethlefoen[3] treated a patch of lupus by repeated freezing with ethyl chloride. This was done at first daily and afterwards every second or third day, the scab that formed being removed each time. In ten weeks the patch was covered with smooth soft skin without any nodules. Reiner[4] still advocates excision in place of newer methods where the skin can be brought together without deformity or where the gap can be filled by transplantation or grafts. The rapidity of the method is a strong point in its favour. Relapses occur under all methods, but in his 52 cases there was no return in 33, and 8 others only required a second operation to complete a cure. E. Stapleton[5] discusses the Finsen light treatment and argues that it it not the light but the inflammatory reaction which leads to the cure, and instances cases of lupus cured by an attack of erysipelas. If the light itself is the active agent it is strange that the face and hands are so often affected. Though some excellent results are attained under this method a few cases, especially those which have undergone previous treatment, are not relieved, and the time required is a serious drawback. X-rays, chemical dressings, and thyroid feeding are desirable as adjuncts, especially as the light cannot be applied to the mucous membranes. Everett Smith[6] obtained a cure in lupus of 15 years' standing by applications of the X-rays every fifth day for 20 minutes. After 12 sittings the skin and conjunctiva were quite healthy. It is not clear how the X-rays influence the disease. Barrett Smith[7] argues that they cause increased phagocytosis; at any rate it has been shown that they do not affect the tubercle bacillus itself directly. The germs of typhoid, cholera, and Malta fever are also uninjured by them or by sunlight, though light quickly destroys the germs of tubercle and those of plague.

**L. Erythematosus.**—Schoonheid[8] in discussing its histology concludes that it is a chronic inflammatory process rather than a granuloma, and that it commences as a perivascular infiltration in the reticular layers of the cutis, due at first to an invasion by leucocytes, and later on of tissue cells. Other changes follow in the epidermis and finally degenerative changes in the elastic fibres and invading cells. The Finsen light treatment seems to be fairly successful, though, owing to the dense tissue, it is difficult to make the area anæmic, which is necessary for success, and to obtain as good results as in L. vulgaris. F. Von Poor[9] sums up the arguments in favour of the tubercular origin of the disease as follows:—It usually occurs in tubercular subjects; it gives a general reaction to tuberculin as well as a local one; giant cells are often found in the lesions; and transitional forms are seen between it and L. vulgaris, as well as tubercular foci in the former disease. Still he thinks himself able to show that none of these arguments are sound, and that the cause of the disease is neither to be found in the action of the tubercle bacillus nor in that of any of its products.

**Drug Eruptions—Arsenic.**—H. G. Brooke and Leslie Roberts[10] analyse the skin affections in the late epidemic, and notice especially the swelling redness and tenderness of the hands and feet, together with numbness and tingling in them, and hyperkeratosis and hyperidriosis. Erythematous rashes elsewhere, or livid blotches, all followed by desquamation and often by pigmentation, or by a peculiar dirty discolouration were common. Herpes, flushing, patches of œdema or cyanosis also occurred. They believe that the action of arsenic is that of overstimulation due to its power, like that of hæmoglobin, for carrying and liberating active oxygen. This tends to an excessive nutrition, at first with over action, and finally with atrophy and degeneration. The theory was first put forward by Binz and Schulz in 1879, and the changes in the tissues can be best explained by such a development of active oxygen in their midst. A. Hall[11] reports a curious case of arsenical pigmentation in an alcoholic woman, which nearly covered and was confined to the abdomen. This was of a uniform brown and peeled in large strips. Elsewhere there was only a slight tint discoverable with fine branny desquamation. Some remarkable results were obtained in a trial of intravenous injections of arsenic for psoriasis at Stockholm.[12] The remedy failed as such, and some symptoms of poisoning appeared. The hæmoglobin was diminished, and the number of leucocytes fell by a half. Albuminuria, pruritus, paræsthesia of the extremities, pigmentation, herpes zoster, and various skin eruptions were noted. Berliner [13] discusses an instance of idiosyncrasy to mercury where most violent suppurative dermatitis and salivation followed the inunction of half a drachm of mercurial ointment daily for 10 days. The urine contained casts, the temperature rose to 102°, and the patient's condition was so bad that she was kept in a permanent warm bath. Such accidents seem much more frequent when mercury is given by the skin or by injection than when administered by the mouth.

**Leprosy.**—C. E. Davis [14] describes his visit to Molokai where 1,073 lepers are segregated and well treated. No remedies have been found effective here. The author is inclined to attribute the introduction of the disease into Hawaii to migrations of oriental nations in the Middle Ages. P. M. May [15] reports a visit to a leper island in the Penrhyn group, where the disease has been introduced about twenty years, and some 29 cases have appeared. Partial segregation has checked its spread, and further measures are to be carried out by the New Zealand Government. Lie's report of the Bergen leper house mentions the failure as remedial agents of Carasguilla's serum, europhen, and Unna's pyrogallol. He found that nearly all nodular lepers throw out numbers of bacilli from the nose and mouth in speaking or sneezing. No attempts to grow them on artificial media were successful. He states that nearly thirty cases of leprosy have been cured at the hospital in question. G. Thin [17] reports two cases of nerve leprosy from his own practice which recovered completely. In the first he employed chaulmoogra oil internally and externally, with nourishing food and an outdoor life; in the second a drachm of gurgun oil was given twice a day and pyrogallic acid ointment was rubbed into the anæsthetic spots. It is remarkable that the spots thus treated regained their normal condition entirely, and he thinks that the pyrogallic acid is able to destroy the leprous bacillus when it has only lodged in the superficial tissues as in this patient, and that the nerve fibres are regenerated. Rapid improvement under small doses of chaulmoogra oil and an ointment of it was also witnessed by Brousse and Vires [18] in a Frenchman who suffered from the nodular form. He had served in Algeria and the disease did not develop till 16 years later. The oil was given in doses of half a drachm daily as larger amounts could not be tolerated.

**Nævus.**—Jutassy [19] reports a most successful case of "port-wine" stain treated by the X-rays. In doing this a too-powerful action was induced in one part and a sore produced which took two months to heal, but the nævus vanished and in eighteen months the skin was normal over the greater part.

**Cancer.**—V. Bie [20] reports Finsen's results with light treatment in 16 cases—7 were cured, 5 improved, 1 relapsed, and 3 were failures. The best cases are small, superficial growths situated where pressure can be applied. F. H. Williams [21] finds that epidermoid cancer, even when too advanced for cure, can be ameliorated and the discharge lessened by treatment with the X-rays. Merrill and Johnson [22] used the rays in six cases with remarkable success, causing the destruction of slow-growing epitheliomas without pain and leaving good healed surfaces. They believe that even if uncurable the pain and rapid progress of superficial carcinoma may be checked by the use of the rays. Dubreuilh and Rocher [23] observed a case of nævus in a woman of 54 years which began to undergo degenerative changes and proved the starting-point of a hæmorrhagic carcinoma requiring excision.

**Blastomycosis.**—Seven cases of this affection of the skin have been recently reported. Dwyer [24] had a female patient showing the eruption on the face and backs of the hands. It was painless, raw, and warty, exuding a yellow fluid when touched. Under increasing doses of iodide and an ointment of iodide of lead, rapid improvement took place Montgomery reports [25] a corn porter who had a rapidly growing tumour on the lip which contained an aërobic fungus and giant cells in the cutis. Iodide of potash had no effect, and the tumour was excised. Another male patient, aged 38, showed lesions on the back of the hands and face which yielded to iodide. In a third case the interscapular region, the left elbow, and a spot below the angle of the left scapula were attacked. Hyde [26] mentions two other patients where the face and forearm respectively were the seat of the disease, and the blastomycetes were found as in the previous cases. He reviews the history of the subject, and finds that males between 50 and 60 are most often affected. There seems no connection with tubercular or syphilitic disease, and the lesions do not recur after excision. Improvement has often followed the use of iodide of potash, but no complete cure has been thus obtained. It differs from tuberculosis verrucosa in this improvement under iodide, in being multiple, and in generally commencing on the face or hands, where probably the infection was first introduced. An instance where the lesions appeared on the left hip, and were confined to that spot, is given by F. G. Harris.[27] The growth went on for four years, and did not reappear after excision. The patient was a woman, and of the advanced age of 78.

**Urticaria.**—A remarkable instance of susceptibility to the action of a special poison is described by L. G. Glover.[28] A hair-wash containing formalin was used by a young married lady, and produced most violent urticaria over nearly the whole body. Not until the lotion bottle was removed from the room did the patient recover. The pathology of urticaria is reviewed by L. Török,[29] who finds that there are no really trustworthy points of difference between angioneuroses and inflammations, and that the changes found in urticaria, erythema multiforme, and E. nodosum are inflammatory in nature. A form of urticaria is sometimes seen in pleurisy, when absorption of the fluid takes place. A case is reported by Gomez,[30] who considers it a favourable sign implying a rapid disappearance of the fluid. The eruption appears most often on the trunk, abdomen, and round the anus. The eruptions of an urticarial type, following the use of diphtheria antitoxin, are considered by Seiffert [31] to be due to embolism. He examined some of the wheals microscopically, and found certain cell-like bodies containing dark included pigment lying in the thrombosed vessels and in the tissues around. He thinks them to be parasitic in nature, but failed to grow them on any media.

**Rhinoscleroma.**—T. V. Marschalko [32] finds that the

Mikulicz cells in this disease are derived from connective-tissue cells, and always in the early stages of the degeneration contain the bacilli; the hyalin, colloid, or fuchsin bodies of Russell, are merely plasma cells degenerated and not parasitic bodies, either in cancer or elsewhere. Thus he is opposed to Unna's view that both the hyalin cells and those of Mikulicz are derived from leucocytes.

**Adiposa Dolorosa.**—This generally attacks women about 50 years of age, and is marked by the increased amount of fat in the subcutaneous tissues, accompanied by much pain. Fatty nodules appear, becoming confluent, pendulous, tender, and painful. There are also headache, paræsthesiæ, and muscular atrophies, with mental weakness. A. Judice[33] reports a fresh case of this rare disease. He found that under thyroid treatment the progress was arrested and great improvement took place.

**Thyroid Treatment.**—Though this substance has to some extent fallen into disuse, J. Galloway[34] points out that the collected reports show that it has great value in special cases. In cutaneous tuberculosis where badly-developed scar tissue and ulceration were prominent it was found particularly useful, though it did not check the progress of active forms of the disease. The constant supervision required where large doses of thyroid are employed is, however, one of the causes of the success obtained in diseases such as lupus, where regularity of treatment is always an important factor. On the other hand, in L. erythematosus, where not much scar tissue is formed, very little good is done by thyroid. In a few cases of psoriasis a quick disappearance of the lesions occurred, and even 10 grains daily of thyroid extract were well borne, but in many others it was quite a failure. The evidence in icthyosis is uncertain, because most of the cases on record have received other treatment as well, such as ointments.

[1] Med. Rec., April 27. [2] Brit. Med. Jour., April 6. [3] Brit. Med. Jour., May 11. [4] Brit. Med. Jour., June 8. [5] Dublin Jour. Med. Sc., March. [6] Brit. Med. Jour., Feb. 9. [7] Brit. Med. Jour., May 4. [8] Brit. Jour. Dermat., April, p. 159. [9] Brit. Jour. Dermat., June. [10] Brit. Jour. Dermat., April. [11] Brit. Jour. Dermat., June. [12] Brit. Med. Jour., Feb. 16. [13] Brit. Jour. Dermat., May. [14] Med. Rec., April 13. [15] Brit. Med. Jour., June 8. [16] Brit. Med. Jour., Jan. 26. [17] Brit. Med. Jour., May. [18] Brit. Jour. Dermat., June. [19] Brit. Med. Jour., May 11. [20] Brit. Med. Jour., Feb. 9. [21] Loc. cit. [22] Loc. cit. [23] Amer. Jour. Med. Sc., April. [24] Jour. Cut. and G. U. Dis., Jan. [25] Loc. cit. [26] Loc. cit. [27] Amer. Jour. Med. Sc., May. [28] Brit. Jour. Dermat., April, p. 153. [29] Ibid., p. 158. [30] Brit. Med. Jour., March 25. [31] Brit. Med. Jour., April 6. [32] Brit. Jour. Dermat., April, p. 157. [33] Brit. Jour. Dermat., May. [34] Edin. Med. Jour., May.

